# Food Insecurity and Depression among US Adults: NHANES 2005–2016

**DOI:** 10.3390/nu14153081

**Published:** 2022-07-27

**Authors:** Nicole Reeder, Terezie Tolar-Peterson, R. Hartford Bailey, Wen-Hsing Cheng, Marion W. Evans

**Affiliations:** 1 Department of Food Science, Nutrition, and Health Promotion, Mississippi State University, Starkville, MS 39762, USA; nr657@msstate.edu (N.R.); wcheng@fsnhp.msstate.edu (W.-H.C.); 2 Pathobiology and Population Medicine, College of Veterinary Medicine, Mississippi State University, Starkville, MS 39762, USA; rhbailey@cvm.msstate.edu; 3 School of Health Professions, The University of Southern Mississippi, Hattiesburg, MS 39406, USA; will.evans@usm.edu

**Keywords:** food insecurity, depression, NHANES, United States

## Abstract

A growing body of evidence suggests that food insecurity is associated with adverse mental health outcomes such as depression and anxiety. In this study, the relationship between food insecurity and depression was examined using data from the 2005–2016 National Health and Nutrition Examination Survey (NHANES). Food insecurity was assessed with the 18-item United States Food Security Survey Module with zero affirmative responses indicating high food security, 1 or 2 affirmative responses indicating marginal food security, and ≥3 affirmative responses indicating food insecurity. Depression was assessed with the Patient Health Questionnaire-9 with scores ≥10 indicating depression. Data were analyzed from 28,448 adult participants aged 20 or older. Food insecurity was present in 19.2% of the sample population (*n* = 5452). Food security status was significantly associated with gender, race, education level, marital status, smoking status, and BMI (Rao-Scott chi-square, *p* < 0.05). Fully food secure and very low food security adults experienced depression at a rate of 5.1% and 25.8%, respectively (Rao-Scott chi-square, *p* < 0.0001). Participants with very low food security had a significantly greater odds of depression than food secure adults, OR = 3.50 (95% CI: 2.98, 4.12). These findings suggest that food insecurity is a significant risk factors for depression in US adults over 20 years of age. To address this issue in our citizenry, police initiatives and public health interventions addressing both food access and mental health should be prioritized.

## 1. Introduction

Food insecurity is the state of having limited or uncertain access to adequate food [1]. Over the past 20 years, the rate of adult food insecurity in the United States (US) has persistently hovered around 10 to 15% of households [1]. Certain populations have significantly higher rates of food insecurity in the US, including households with young children, non-Hispanic Black households, and Hispanic households [1]. Food insecurity is closely tied to poverty, and the persistence of food insecurity is related to under- and unemployment, high housing costs relative to income, lack of access to food assistance programs, and high medical and healthcare costs [2]. Individuals living just above the poverty line can experience unexpected life events, i.e., home or car repair, unexpected medical diagnoses, or the loss of employment which can cause temporary food insecurity. People in these situations can suddenly become faced with decisions of spending their limited financial resources on food or on other unplanned immediate expenses.

Research has shown that individuals who are food insecure are at an increased risk of developing chronic diseases and spend an additional $1863 per year on medical expenses compared to food secure individuals, equating to an additional $77.5 billion in healthcare expenditures annually [3,4]. Food insecurity has been linked to an increased risk of cardiovascular disease, type 2 diabetes, hypertension, dyslipidemia, and metabolic syndrome, making food insecurity a critical public health issue [4,5,6,7,8,9,10,11]. In addition to physical health, mental health disorders such as depression are also increasingly being linked to food insecurity. Food insecurity and depression have been found to coexist among individuals living with or at risk for HIV/AIDS [12,13,14], college students [15,16,17,18,19], and women who are pregnant or mothers of young children [20,21,22,23,24].

Clinical depression is different from sadness and normal day to day fluctuations in mood and is diagnosed based on experiencing a depressed mood or loss of interest in activities for at least two weeks [25]. Individuals with clinical depression will also commonly experience weight gain or weight loss, insomnia, or hypersomnia, being noticeably restless or slow, fatigue, feelings of worthlessness, decreased concentration, and/or thoughts of death or suicide [25]. Depression is one of the largest contributors to disability globally [26] and affects about 8% of adults in the US [27,28]. Individuals living in poverty are disproportionally affected by depression, and women who have household incomes less than 100% of the federal poverty level (FPL) have the highest rates of depression in the US [27]. While rates of depression and food insecurity are both higher among individuals living in poverty, there is some evidence that food insecurity is associated with mental health independent of poverty level. Research has indicated that food insecure adults with household incomes at or below 130% of the FPL have greater odds of depression than food secure adults who are at or below 130% of the FPL [29]. Among older adults, food insecurity has also been shown to be a stronger predictor of depressive symptoms than financial difficulty, having an unmet health need, or having inadequate housing [30].

A link between food insecurity and depression fundamentally makes sense. Individuals with food insecurity may feel stress from not knowing where the next meal will come from, shame or alienation from relying on food assistance, or guilt from acquiring food in socially unacceptable ways. Individuals with food insecurity may also have less social support, which is another risk factor for depression [31]. It remains unknown, however, how generalizable the association between food insecurity and depression is among US adults, and how much of this link may be attributed to poverty or other factors. The objective of this study was thus to examine whether food insecurity and depression are associated among a nationally representative sample of U.S. adults, using a wide range of data available from the National Health and Nutrition Examination Survey.

## 2. Methods

### 2.1. Study Population

**Figure 1:**
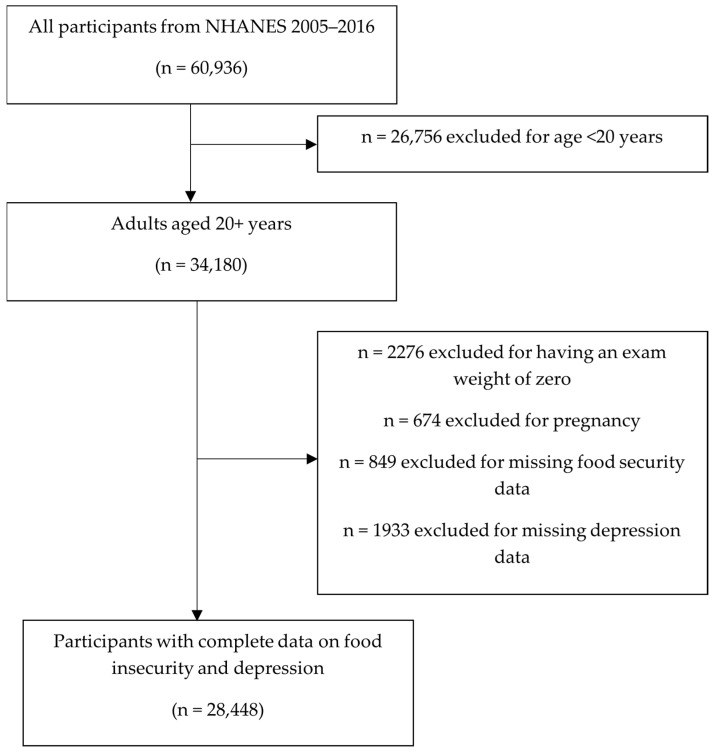
Flow chart of inclusion and exclusion criteria for the study sample.

The National Health and Nutrition Examination Survey (NHANES) is a cross-sectional, population-based study of the non-institutionalized, civilian population in the US that assesses the health and nutritional status of adults and children [32]. Data collection for NHANES consists of an in-home interview portion and a physical exam portion which takes place at a mobile examination center. The interview consists of demographic, socioeconomic, dietary, and health-related questions, and the physical exam portion includes medical, dental, physiological and laboratory measurements. For this analysis, data were drawn from the 2005 to 2006 cycle through the most current cycle with food security data available, the 2015 to 2016 cycle. Inclusion criteria for the analysis sample were adults ages 20+ with complete data on food insecurity and depression measurements. The initial eligible sample for this analysis was 34,180 participants aged 20 and older. Among this sample, participants were excluded for the following reasons: exam weight of zero (*n* = 2276), pregnancy (*n* = 674), missing food security data (*n* = 849), and missing depression data (*n* = 1933). This resulted in a final sample of 28,448 adults (Figure 1).

The National Center for Health Statistics Review Board approved NHANES, and all NHANES participants provided written informed consent. Due to the de-identified nature of NHANES data, the conduction of this secondary data analysis was determined to be exempt from human subjects research approval by the Mississippi State University Institutional Review Board.

### 2.2. Food Insecurity

Household food insecurity was measured using the US Food Security Survey Module (US FSSM). The US FSSM is a validated survey that is considered the gold standard for measuring food insecurity in the US [33,34]. Beginning with the 2005 to 2006 data collection cycle, all households were administered these questions and no income screener was used. The US FSSM contains 10 questions for households without children and eight additional questions for households with children. Based on a household’s responses to these surveys, the USDA has defined four levels of food security under which households can be characterized. These are: (1) full, or high, food security (FFS), where the household has no problems with or anxiety about obtaining adequate food, (2) marginal food security (MFS), where the household occasionally had problems or anxiety about obtaining adequate food, but did not have to alter the quality, variety, or quantity of food they ate, (3) low food security (LFS), where the household had to reduce the quality, variety, or desirability of the food they ate, but did not have to change the quantity of food they ate, and (4) very low food security (VLFS), where at least one member of the household had their eating patterns disrupted and food intake reduced due to a lack of money for food [35]. Food insecurity survey scores were classified as FFS if zero affirmative responses are reported, MFS for 1 or 2 affirmative responses, LFS for 3 to 5 affirmative responses, and VLFS for 6 to 10 affirmative responses [36]. The four food security categories were collapsed into a binary variable for some analyses with those who were classified as having FFS or MFS being considered “food secure” and those with LFS or VLFS being considered “food insecure.”

### 2.3. Depression

Depression was assessed using the Patient Health Questionnaire-9 (PHQ-9), which was administered to all NHANES participants ages 12 and over as part of the computer assisted personal interview in the mobile exam center. The PHQ-9 is a self-report depression screening tool that is based on the nine items reflective of major depressive disorder in the Diagnostic and Statistical Manual of Mental Disorders, Fourth Edition [37]. It is a reliable and valid measure of depression severity that asks questions about the frequency of depressive symptoms experienced over the past two weeks. Each question is scored from “0” (not at all) to “3” (nearly every day) with the final questionnaire scores ranging from 0–27. Scores of 10 or greater were used to denote the presence of depression [37].

### 2.4. Covariates

Covariates include age, sex, race (non-Hispanic white, non-Hispanic black, Hispanic, and “other”), family poverty-to-income ratio (PIR) (0–130% FPL, 131–185% FPL, 186–300% FPL, and >300% FPL), body mass index (BMI) (<25, 25–29.9, and ≥30), marital status (married or living with a partner, never married, or separated, widowed, or divorced) educational attainment (<high school, high school diploma/GED, some college/associates degree, college graduate), and smoking status (current smoker—smoked > 100 cigarettes in lifetime and smoke at time of survey; past smoker—smoked > 100 cigarettes in lifetime, but not smoking at time of survey; never smoker—smoked < 100 cigarettes in lifetime) [38].

Family poverty-to-income ratio cut off points are based on common cut points of interest related to food insecurity. Income eligibility for Supplemental Nutrition Assistance Program (SNAP) benefits is ≤130% of the FPL, which denotes the first group of those households with incomes 0–130% of the FPL. The next group is households with incomes not eligible for SNAP, but who would still qualify for participation in the Women, Infants, and Children (WIC) program and National School Lunch Program (NSLP), which both have eligibility cut offs of incomes ≤185% of the FPL. Finally, while food insecurity still occurs to some extent among households with incomes 186–300% of the FPL, food insecurity is rare above 300% of the FPL [1] and thus the last two family poverty-to-income ratio groups are coded accordingly.

### 2.5. Statistical Analysis

Twelve-year survey weights were calculated and used in all analyses to adjust for unequal selection probability and non-response bias in accordance with NHANES analytical guidelines [39]. Mobile exam center weights were used and re-calculated to reflect the probability of being sampled in the 12-year period. Descriptive statistics were calculated to summarize sociodemographic characteristics across different food security categories. Differences in participant characteristics across the four categories of household food security (FFS, MFS, LFS, and VLFS) were examined using univariate linear regression models for continuous variables and chi-square tests for categorical variables. The relationship between food insecurity and depression was examined using a series of multivariate logistic regression models. Before testing logistic regression models, Spearman correlation coefficients were run to check for collinearity, and correlation coefficients >0.9 were considered to indicate collinearity. The first model adjusted for poverty-to-income ratio, the second model adjusted for poverty-to-income ratio, age, race, and gender, and the final model adjusted for poverty-to-income ratio, age, race, gender, marital status, education, smoking, and BMI. Effect modification between food security status, gender, and race/ethnicity was also tested by product interaction terms in the fully adjusted logistic regression models.

Next, backwards stepwise selection was used to fit a logistic regression model to identify risk factors associated with depression for individuals with food insecurity. All analyses were conducted using SAS Version 9.4 (SAS Institute, Cary, NC, USA). All tests were two-tailed and the level of significance (α) was set at 0.05.

## 3. Results

A total of 28,448 individuals were included in the final data analysis. The prevalence of food insecurity in this group (those with LFS and VLFS) was 19.2%. Food security status was significantly associated with gender, race, education, family PIR, marital status, smoking status, and BMI (Table 1). Adults with food insecurity were more likely to be non-White, have a lower educational attainment, have a lower poverty-to-income ratio, be single, widowed, divorced, or separated, be smokers, and have obesity. Among individuals with VLFS, just 8% were college graduates, though 35% had attended at least some college. Fifty-five percent of adults with food insecurity had incomes ≤130% of the federal poverty level; however, even in the VLFS group, 24.2% of adults reported incomes that were >185% of the federal poverty level. The proportion of individuals who are smokers was greatest among the most food insecure participants (44% of those with VLFS compared to 17% of those with FFS). As an indication of depression, 8.8% of the study population had PHQ-9 scores ≥10. There was a significant difference in prevalence of depression across food security categories (*p* < 0.0001), with 5.1% of FFS adults experiencing depression compared to 25.8% of adults with VLFS.

Food insecurity was associated with a greater odds of depression in a dose–response pattern, as shown in Table 2. After adjustment for family poverty-to-income ratio (Model 1), compared to participants with FFS, participants with VLFS had greater odds of having depression, OR = 4.11 (95% CI: 3.52, 4.80). This association persisted after further adjusting for age, race, and gender (Model 2) and marital status, educational attainment, smoking status, and BMI (Model 3). In the fully adjusted model (Model 3), the OR (95% CIs) were 1.60 (1.27, 2.02), 1.88 (1.58, 2.24), and 3.50 (2.98, 4.12) for participants with MFS, LFS, and VLFS, respectively. There were no significant interaction effects found between food security status and gender (*p* = 0.161) nor food security status and race (*p* = 0.362). No collinearity was detected between variables.

Finally, a sub-analysis of only adults with food insecurity (*n* = 5452) was conducted, in which a multivariate logistic regression model was fit to examine risk factors for depression among food insecure adults. A manual backward selection process was followed for variable selection, in which after fitting the model, the variable with the greatest *p*-value was removed and the model refit. This was continued until the final model contained only variables with a *p*-value of less than 0.05. The first variable removed was age (*p* = 0.198), followed by educational attainment (*p* = 0.096). The final model included gender, race, PIR, marital status, smoking status, and BMI. Among adults with food insecurity (those with LFS and VLFS), odds of having depression were higher for women, those of non-Hispanic White race/ethnicity, those with incomes less than 130% of the federal poverty level, adults who are widowed, divorced, or separated, adults who smoke, and adults with obesity (Table 3). The odds of having depression were 68% higher for women with food insecurity compared to men with food insecurity, and food insecure individuals who smoke had a more than two-fold greater odds of having depression compared to food insecure individuals who never smoked. By race, non-Hispanic Black and Hispanic participants with food insecurity had lower odds of having depression than non-Hispanic White participants, OR = 0.80 (95% CI: 0.64, 0.99) and OR = 0.68 (95% CI: 0.54, 0.86), respectively.

## 4. Discussion

In this nationally representative sample of US adults, food insecurity was significantly associated with an increased odds of having depression. Among US adults with food insecurity, being female, widowed, divorced, or separated, smoking cigarettes, having a household income <130% of the FPL, and having obesity all increased the odds of experiencing depression. Moreover, while rates of food insecurity were higher among non-Hispanic Black and Hispanic participants compared to non-Hispanic White participants, non-Hispanic White participants who were food insecure were more likely to have depression than non-Hispanic Black or Hispanic participants who were food insecure. These findings ultimately provide further evidence that the consequences of food insecurity have a significant impact on well-being.

One of the strongest predictors of depression for food insecure adults was cigarette smoking. Tobacco use is inversely associated with socioeconomic status [40], and adults with low socioeconomic status are more likely to have started smoking at a younger age and smoke for a longer duration of their life [41]. Bearing that in mind, food insecure adults who smoke may be more likely to be among the US adults that consistently struggle with food insecurity, rather than be adults transiently experiencing food insecurity. Food insecurity is also an inherently stressful state and food insecure adults struggling with depression, stress, or anxiety may turn to unhealthy coping behaviors such as the use of tobacco or alcohol for stress relief. Finally, monetary resources that are used for purchasing cigarettes may displace monetary resources that could otherwise be used for purchasing food. Another factor that significantly predicted whether an adult with food insecurity had depression was marital status. Food insecure adults who are widowed, divorced, or separated are likely to have less social and financial support than they once had, placing them at greater risk for food insecurity and for social isolation that would increase the risk for depression. Findings related to race were similar to established trends in depression rates by race in the US. Whites generally have a higher lifetime prevalence of major depressive disorder than African Americans [42] and this trend persisted when assessing a subpopulation of adults who all reported having food insecurity. Finally, we found that gender was one of the most significant risk factors for depression among food insecure adults. Women with food insecurity were 68% more likely to report experiencing depression than men with food insecurity. While women generally experience depression at greater rates than men [27], women also suffer the brunt of the consequences of food insecurity. Part of this is related to household structure, as women are more often in charge of managing food in a household [43], and women tend to shield other household members, especially children, from the consequences of food insecurity [44,45].

Our results suggest that food insecurity and depression commonly coexist, and as food insecurity becomes more severe, depression also becomes more severe. These findings confirm similar findings from other studies that have suggested a dose–response relationship between food insecurity and depression. For example, among mothers of 3-year-old children across 18 major US cities, the percentage of mothers with anxiety or depression jumps from 16.9% to 21.0% to 30.3% between mothers who are fully food secure, marginally food secure, and food insecure [46]. The SARS-CoV2 pandemic presented a unique opportunity to study this relationship further, as job losses, lock downs, and social isolation led to an increasing number of people struggling with food insecurity and mental illness worldwide. One study found that during the SARS-CoV2 pandemic, adults with incomes <200% of the federal poverty level who were food insecure had 3.5 times higher odds of experiencing depression compared to adults without food insecurity, and 3.6 times higher odds of anxiety [47]. Another study found that during the pandemic, the odds of having depression were 2.42, 3.84, and 7.75 times higher for adults with MFS, LFS, and VLFS, respectively [48], which provides further evidence for a dose–response relationship between food insecurity and depression.

This current study has several limitations which should be noted. First, this study is a secondary data analysis of cross-sectional data, and thus, causation cannot be established. Second, food insecurity is a self-reported measure based on participant’s responses to the US Food Security Survey Module, which may add bias due to variability in an individual’s perceptions of their degree of food security. This survey also aims to assess food insecurity at the household level rather than at the individual level which creates the potential for a household member to be misclassified as food insecure or food secure if their food security status differs from that of another household member; though, degree of food security is generally similar for all members of a household [33]. Finally, food insecurity is complexly related to many other social determinants of health, and it is not possible for this study to account for all possible confounding factors that may affect the relationship between food insecurity and adverse health outcomes such as depression.

## 5. Conclusions

Despite the limitations stated above, this study is significant because it provides further evidence of the burden of food insecurity in the US and how it can contribute to the mental health crisis being experienced. Approximately 10–15% of US households are persistently food insecure each year, and these adults have a much higher risk of mental health disorders. Food assistance programs such as SNAP and WIC that aim to alleviate some of the burden of food insecurity may help decrease the risk of depression among low-income adults [29], but they are not enough to eliminate the concurrent burden of mental illness. Certain adults with food insecurity also may need access to mental health resources, and research suggests that this population is reaching out for help. Data from 2007–2014 indicate that adults with food insecurity are more likely to have seen a mental health professional in the past year than adults without food insecurity [49]. Mental health professionals can assist by considering asking their patients if they are struggling with food availability, and if so, referring them out to relevant food assistance programs. Likewise, food banks or other non-profit organizations that offer nutrition education programming could consider also providing basic programming on healthy coping behaviors when experiencing stress, anxiety, or depression. Public health interventions should be targeted and focused on populations at heighted risk for both food insecurity and depression, such as women, households with incomes ≤130% of the federal poverty level, and adults who are newly widowed, divorced, or separated. Finally, given the strong body of evidence supporting a dose–response relationship between food insecurity and depression in the US, public and private resources that address both food access and mental health should be prioritized.

## Figures and Tables

**Table 1:** Household food security and sociodemographic characteristics: National Health and Nutrition Examination Surveys 2005–2016.

Characteristic	Full Food Security (*n* = 19,879)	SE	Marginal Food Security (*n* = 3117)	SE	Low Food Security (*n* = 3415)	SE	Very Low Food Security (*n* = 2037)	SE	*p* ^c^
Age, y ^a^	49.1	0.3	43.0	0.5	41.6	0.4	42.5	0.5	<0.0001
Gender ^b^									0.0322
Male	49.7	0.3	46.9	0.9	47.8	1.0	48.8	1.7	
Female	50.3	0.3	53.1	0.9	52.2	1.0	51.2	1.7	
Race/ethnicity ^b^									<0.0001
Non-Hispanic White	75.0	1.2	50.0	2.5	45.3	2.4	53.5	2.5	
Non-Hispanic Black	8.9	0.6	16.7	1.4	17.9	1.4	19.4	1.5	
Hispanic	9.4	0.7	26.2	2.2	30.1	2.0	20.8	1.8	
Other	6.6	0.4	7.1	0.8	6.7	0.7	6.3	0.8	
Education ^b^									<0.0001
<High school	12.2	0.6	25.7	1.4	35.4	1.2	29.8	1.5	
High school graduate	21.5	0.6	27.4	1.0	25.7	1.0	27.6	1.4	
Some college	31.4	0.6	34.1	1.3	30.8	1.3	34.6	1.3	
College graduate or above	34.8	1.1	12.8	1.0	8.0	0.9	8.0	1.2	
Poverty-to-income ratio (family) ^b^									<0.0001
≤130% FPL	12.9	0.6	39.0	1.8	52.9	1.6	59.7	2.1	
130–185% FPL	8.5	0.3	18.4	1.2	19.1	1.1	16.1	1.3	
186–300% FPL	17.9	0.5	23.9	1.5	16.7	1.2	15.7	1.3	
>300% FPL	60.7	1.0	18.7	1.5	11.3	1.3	8.5	1.3	
Marital Status ^b^									<0.0001
Never married	16.1	0.6	20.6	1.2	23.4	1.3	26.1	1.6	
Married or living with partner	66.6	0.7	57.3	1.4	54.9	1.5	44.8	1.9	
Widowed, divorced, or separated	17.3	0.4	22.1	1.0	21.7	1.0	29.1	1.5	
Smoking Status ^b^									<0.0001
Current smoker	17.0	0.5	28.7	1.3	33.3	1.4	44.0	1.8	
Past smoker	26.7	0.5	21.6	1.1	18.1	0.9	16.9	1.2	
Never smoker	56.3	0.7	49.7	1.5	48.6	1.4	39.1	1.5	
BMI ^b^									<0.0001
Normal weight (<25)	32.0	0.6	28.1	1.1	26.9	1.1	30.6	1.6	
Overweight (25–29.9)	33.4	0.5	31.6	1.2	29.8	1.1	26.7	1.2	
Obese (≥30)	34.6	0.6	40.3	1.2	43.3	1.2	42.8	1.6	
Depression ^b^									<0.0001
PHQ-9 Score ≥10	5.1	0.2	11.5	0.8	14.7	0.9	25.8	1.4	

SE, standard error; FPL, federal poverty level; BMI, body mass index. ^a^ Age is mean (SE); ^b^ Categorical variables are weighted % (SE); ^c^ *p*-values from Rao-Scott F-adjusted chi-square tests for categorical variables and analysis of variance for continuous variables.

**Table 2:** Odds of having depression by food security status.

	Depression, n	Crude Model, OR	95% CI	Model 1, OR	95% CI	Model 2, OR	95% CI	Model 3, OR	95% CI
Full food security	1157	Ref.		Ref.		Ref.		Ref.	
Marginal food security	334	2.44	1.99–2.99	1.71	1.37–2.13	1.84	1.46–2.31	1.60	1.27–2.02
Low food security	491	3.23	2.75–3.80	2.05	1.73–2.44	2.24	1.85–2.71	1.88	1.58–2.24
Very low food security	524	6.51	5.61–7.55	4.11	3.52–4.80	4.44	3.77–5.22	3.50	2.98–4.12

OR, odds ratio; CI, confidence interval. Crude model is unadjusted. Model 1 is adjusted for family poverty-to-income ratio. Model 2 is adjusted for poverty-to-income ratio, age, race, and gender. Model 3 is adjusted for poverty-to-income ratio, age, race, gender, marital status, educational attainment, smoking status, and BMI.

**Table 3:** Model fitting for odds of depression among food insecure individuals.

Effect	Level	Odds Ratio	95% CI	*p*
Gender	Female vs. Male	1.68	1.41–2.00	<0.0001
Race	Non-Hispanic Black vs. Non-Hispanic White	0.80	0.64–0.99	0.042
	Hispanic vs. Non-Hispanic White	0.68	0.54–0.86	0.002
	Other vs. Non-Hispanic White	0.98	0.68–1.41	0.906
PIR	130–185% FPL vs. ≤130% FPL	0.77	0.60–0.97	0.029
	186–300% FPL vs. ≤130% FPL	0.59	0.43–0.81	0.001
	>300% FPL vs. ≤130% FPL	0.50	0.32–0.77	0.002
Marital status	Married or living with partner vsNever married	0.91	0.73–1.14	0.409
	Widowed, divorced, or separated vsNever married	1.65	1.30–2.09	<0.0001
Smoking Status	Past smoker vs. Never smoker	1.35	1.00–1.84	0.054
	Current smoker vs. Never smoker	2.11	1.73–2.59	<0.0001
BMI	Overweight (25–29.9) vs. Normal weight	1.09	0.83–1.43	0.538
	Obese (≥30) vs. Normal weight	1.49	1.17–1.88	0.001

CI, confidence interval; PIR, family poverty-to-income ratio; FPL, federal poverty level; BMI, body mass index.

## Data Availability

NHANES data are publicly available from https://www.cdc.gov/nchs/nhanes/index.htm accessed on 20 April 2021.

## References

[1] Coleman-Jensen A., Rabbitt M.P., Gregory C.A., Singh A. (2021). Household Food Security in the United States in 2020, ERR-298. www.ers.usda.gov.

[2] The U.S. Conference of Mayors (2016). Report on Hunger and Homelessness.

[3] Berkowitz S.A., Basu S., Meigs J.B., Seligman H.K. (2018). Food insecurity and health care expenditures in the United States, 2011–2013. Health Serv. Res..

[4] Seligman H.K., Laraia B.A., Kushel M.B. (2010). Food insecurity is associated with chronic disease among low-income NHANES participants. J. Nutr..

[5] Sun Y., Liu B., Rong S., Du Y., Xu G., Snetselaar L.G., Wallace R.B., Bao W. (2020). Food insecurity is associated with cardiovascular and all-cause mortality among adults in the United States. J. Am. Heart Assoc..

[6] Holben D.H., Pheley A.M. (2006). Diabetes risk and obesity in food-insecure households in rural Appalachian Ohio. Prev. Chronic Dis..

[7] Fitzgerald N., Hromi-Fiedler A., Segura-Pérez S., Pérez-Escamilla R. (2011). Food insecurity is related to increased risk of type 2 diabetes among Latinas. Ethn. Dis..

[8] Irving S.M., Njai R.S., Siegel P.Z. (2014). Food insecurity and self-reported hypertension among Hispanic, black, and white adults in 12 states, Behavioral Risk Factor Surveillance System, 2009. Prev. Chronic Dis..

[9] Myers C.A., Martin C.K., Newton R.L., Apolzan J.W., Arnold C.L., Davis T.C., Price-Haywood E.G., Katzmarzyk P.T. (2019). Cardiovascular health, adiposity, and food insecurity in an underserved population. Nutrients.

[10] Tayie F.A., Zizza C.A. (2009). Food insecurity and dyslipidemia among adults in the United States. Prev. Med..

[11] Parker E.D., Widome R., Nettleton J.A., Pereira M.A. (2010). Food security and metabolic syndrome in U.S. adults and adolescents: Findings from the National Health and Nutrition Examination Survey, 1999–2006. Ann. Epidemiol..

[12] Kapulsky L., Tang A.M., Forrester J.E. (2015). Food insecurity, depression, and social support in HIV-infected Hispanic individuals. J. Immigr. Minor. Health.

[13] Tsai A.C., Bangsberg D.R., Frongillo E.A., Hunt P.W., Muzoora C., Martin J.N., Weiser S.D. (2012). Food insecurity, depression and the modifying role of social support among people living with HIV/AIDS in rural Uganda. Soc. Sci. Med..

[14] Davey-Rothwell M.A., Flamm L.J., Kassa H.T., Latkin C.A. (2014). Food insecurity and depressive symptoms: Comparison of drug using and nondrug using women at risk for HIV. J. Community Psychol..

[15] Reeder N., Tapanee P., Persell A., Tolar-peterson T. (2020). Food insecurity, depression, and race: Correlations observed among college students at a university in the Southeastern United States. Int. J. Environ. Res. Public Health.

[16] Payne-Sturges D.C., Tjaden A., Caldeira K.M., Vincent K.B., Arria A.M. (2018). Student hunger on campus: Food insecurity among college students and implications for academic institutions. Am. J. Health Promot..

[17] Becerra M.B., Becerra B.J. (2020). Psychological distress among college students: Role of food insecurity and other social determinants of mental health. Int. J. Environ. Res. Public Health.

[18] Raskind I.G., Haardörfer R., Berg C.J. (2019). Food insecurity, psychosocial health and academic performance among college and university students in Georgia, USA. Public Health Nutr..

[19] Wattick R.A., Hagedorn R.L., Olfert M.D. (2018). Relationship between diet and mental health in a young adult Appalachian college population. Nutrients.

[20] Lent M.D., Petrovic L.E., Swanson J.A., Olson C.M. (2009). Maternal mental health and the persistence of food insecurity in poor rural families. J. Health Care Poor Underserved.

[21] Hromi-Fiedler A., Bermúdez-Millán A., Segura-Pérez S., Pérez-Escamilla R. (2011). Household food insecurity is associated with depressive symptoms among low-income pregnant Latinas. Matern. Child Nutr..

[22] Huddleston-Casas C., Charnigo R., Simmons L.A. (2009). Food insecurity and maternal depression in rural, low-income families: A longitudinal investigation. Public Health Nutr..

[23] Laraia B.A., Siega-Riz A.M., Gundersen C., Dole N. (2006). Psychosocial factors and socioeconomic indicators are associated with household food insecurity among pregnant women. J. Nutr..

[24] Cook J.T., Black M., Chilton M., Cutts D., de Cuba S.E., Heeren T.C., Rose-Jacobs R., Sandel M., Casey P.H., Coleman S. (2013). Are food insecurity’s health impacts underestimated in the U.S. population? Marginal food security also predicts adverse health outcomes in young U.S. children and mothers. Adv. Nutr..

[25] American Psychiatric Association (2020). Diagnostic and Statistical Manual of Mental Disorders.

[26] World Health Organization (2017). Depression and Other Common Mental Disorders: Global Health Estimates.

[27] Brody D.J., Pratt L.A., Hughes J.P. (2018). Prevalence of Depression among Adults Aged 20 and Over: United States, 2013–2016. NCHS Data Brief.

[28] Hasin D.S., Sarvet A.L., Meyers J.L., Saha T.D., Ruan W.J., Stohl M., Grant B.F. (2018). Epidemiology of adult DSM-5 major depressive disorder and its specifiers in the United States. JAMA Psychiatry.

[29] Leung C.W., Epel E.S., Willett W.C., Rimm E.B., Laraia B.A. (2015). Household food insecurity is positively associated with depression among low-income Supplemental Nutrition Assistance Program participants and income-eligible nonparticipants. J. Nutr..

[30] Johnson C.M., Sharkey J.R., Wesley R. (2011). Indicators of material hardship and depressive symptoms among homebound older adults living in North Carolina. J. Nutr. Gerontol. Geriatr..

[31] Kollannoor-Samuel G., Wagner J., Damio G., Segura-Pérez S., Chhabra J., Vega-López S., Pérez-Escamilla R. (2011). Social support modifies the association between household food insecurity and depression among Latinos with uncontrolled type 2 diabetes. J. Immigr. Minor. Health.

[32] NHANES—About the National Health and Nutrition Examination Survey. https://www.cdc.gov/nchs/nhanes/about_nhanes.htm.

[33] Bickel G., Nord M., Price C., Hamilton W., Cook J. (2000). Measuring Food Security in the United States: Guide to Measuring Household Food Security. https://naldc.nal.usda.gov/download/38369/PDF.

[34] Carlson S.J., Andrews M.S., Bickel G.W. (1999). Measuring food insecurity and hunger in the United States: Development of a national benchmark measure and prevalence estimates. J. Nutr..

[35] United States Department of Agriculture Economic Research Service Definitions of Food Security. https://www.ers.usda.gov/topics/food-nutrition-assistance/food-security-in-the-us/definitions-of-food-security/.

[36] United States Department of Agriculture Economic Research Service (2012). U.S. Adult Food Security Survey Module: Three-stage design, with screeners. https://www.ers.usda.gov/media/8279/ad2012.pdf.

[37] Kroenke K., Spitzer R.L., Williams J.B.W. (2001). The PHQ-9: Validity of a brief depression severity measure. J. Gen. Intern. Med..

[38] Centers for Disease Control and Prevention NHIS—Adult Tobacco Use—Glossary. https://www.cdc.gov/nchs/nhis/tobacco/tobacco_glossary.htm.

[39] Centers for Disease Control and Prevention, National Center for Health Statistics (2018). National Health and Nutrition Examination Survey: Analytic Guidelines, 2011–2014 and 2015–2016.

[40] Kim-Mozeleski J.E., Pandey R. (2020). The intersection of food insecurity and tobacco use: A scoping review. Health Promot. Pract..

[41] Siahpush M., Singh G.K., Jones P.R., Timsina L.R. (2010). Racial/ethnic and socioeconomic variations in duration of smoking: Results from 2003, 2006 and 2007 Tobacco Use Supplement of the Current Population Survey. J. Public Health.

[42] Bailey R.K., Mokonogho J., Kumar A. (2019). Racial and ethnic differences in depression: Current perspectives. Neuropsychiatr. Dis. Treat..

[43] Taillie L.S. (2018). Who’s cooking? Trends in US home food preparation by gender, education, and race/ethnicity from 2003 to 2016. Nutr. J..

[44] McIntyre L., Glanville N.T., Raine K.D., Dayle J.B., Anderson B., Battaglia N. (2003). Do low-income lone mothers compromise their nutrition to feed their children?. CMAJ.

[45] Nord M. (2013). Youth are less likely to be food insecure than adults in the same household. J. Hunger Environ. Nutr..

[46] Whitaker R.C., Phillips S.M., Orzol S.M. (2006). Food insecurity and the risks of depression and anxiety in mothers and behavior problems in their preschool-aged children. Pediatrics.

[47] Fang D., Thomsen M.R., Nayga R.M. (2021). The association between food insecurity and mental health during the COVID-19 pandemic. BMC Public Health.

[48] Wolfson J.A., Garcia T., Leung C.W. (2021). Food insecurity is associated with depression, anxiety, and stress: Evidence from the early days of the COVID-19 Pandemic in the United States. Health Equity.

[49] Burruss N.C., Girgis M., Green K.E., Lu L., Palakshappa D. (2021). Association between food insecurity and access to a mental health professional: Cross-sectional analysis of NHANES 2007–2014. BMC Public Health.

